# Increased Expression of Viral Sensor MDA5 in Pancreatic Islets and in Hormone-Negative Endocrine Cells in Recent Onset Type 1 Diabetic Donors

**DOI:** 10.3389/fimmu.2022.833141

**Published:** 2022-03-11

**Authors:** Laura Nigi, Noemi Brusco, Giuseppina E. Grieco, Daniela Fignani, Giada Licata, Caterina Formichi, Elena Aiello, Lorella Marselli, Piero Marchetti, Lars Krogvold, Knut Dahl Jorgensen, Guido Sebastiani, Francesco Dotta

**Affiliations:** ^1^ Diabetes Unit, Department of Medicine, Surgery and Neurosciences, University of Siena, Siena, Italy; ^2^ Fondazione Umberto Di Mario, c/o Toscana Life Sciences, Siena, Italy; ^3^ Department of Clinical and Experimental Medicine, University of Pisa, Pisa, Italy; ^4^ Paediatric Department, Oslo University Hospital, Oslo, Norway; ^5^ Faculty of Medicine, University of Oslo, Oslo, Norway; ^6^ Tuscany Centre for Precision Medicine (CReMeP), Siena, Italy

**Keywords:** pancreas, pancreatic islet, type 1 diabetes, innate immunity, viral sensor, enteroviruses, MDA5, dedifferentiation

## Abstract

The interaction between genetic and environmental factors determines the development of type 1 diabetes (T1D). Some viruses are capable of infecting and damaging pancreatic β-cells, whose antiviral response could be modulated by specific viral RNA receptors and sensors such as melanoma differentiation associated gene 5 (MDA5), encoded by the IFIH1 gene. MDA5 has been shown to be involved in pro-inflammatory and immunoregulatory outcomes, thus determining the response of pancreatic islets to viral infections. Although the function of MDA5 has been previously well explored, a detailed immunohistochemical characterization of MDA5 in pancreatic tissues of nondiabetic and T1D donors is still missing. In the present study, we used multiplex immunofluorescence imaging analysis to characterize MDA5 expression and distribution in pancreatic tissues obtained from 22 organ donors (10 nondiabetic autoantibody-negative, 2 nondiabetic autoantibody-positive, 8 recent-onset, and 2 long-standing T1D). In nondiabetic control donors, MDA5 was expressed both in α- and β-cells. The colocalization rate imaging analysis showed that MDA5 was preferentially expressed in α-cells. In T1D donors, we observed an increased colocalization rate of MDA5-glucagon with respect to MDA5-insulin in comparison to nondiabetic controls; such increase was more pronounced in recent-onset with respect to long-standing T1D donors. Of note, an increased colocalization rate of MDA5-glucagon was found in insulin-deficient-islets (IDIs) with respect to insulin-containing-islets (ICIs). Strikingly, we detected the presence of MDA5-positive/hormone-negative endocrine islet-like clusters in T1D donors, presumably due to dedifferentiation or neogenesis phenomena. These clusters were identified exclusively in donors with recent disease onset and not in autoantibody-positive nondiabetic donors or donors with long-standing T1D. In conclusion, we showed that MDA5 is preferentially expressed in α-cells, and its expression is increased in recent-onset T1D donors. Finally, we observed that MDA5 may also characterize the phenotype of dedifferentiated or newly forming islet cells, thus opening to novel roles for MDA5 in pancreatic endocrine cells.

## 1 Introduction

Type 1 diabetes mellitus (T1D) is a chronic autoimmune disease characterized by the progressive destruction of pancreatic β-cells by the immune system, leading to the absolute loss of insulin secretory function. Although molecular mechanisms involved in T1D pathogenesis are still under investigation, disease progression and onset have been widely recognized as the result of the interaction between a predisposing genetic background and environmental factors that may trigger β-cell destruction and immune system activation (1). Among identified environmental factors, viral infections have been undoubtedly shown to play a prominent role (2, 3). As a matter of fact, multiple evidences of *Enterovirus* infection of pancreatic islets in T1D donors have now been confirmed by several studies, also adopting rigorous methodological cross-validation approaches in various patient cohorts (4–10).

The susceptibility of β-cells to viral infections and the activation of their innate immunity upon viral invasion are crucial factors for the subsequent inflammatory response of islet cells (11). Indeed, we and others have previously shown that one of the isoforms of the main Coxsackieviruses entry receptor, namely Coxsackie Adenovirus Receptor (CAR), is preferentially expressed in β-cells in human pancreas, thus conferring specificity and vulnerability of β-cells to certain viruses (12). In addition, it has been recently confirmed that the expression of several markers of interferon (IFN) signature (e.g., MxA, PKR, and HLA-I) in the pancreatic islets of T1D and of islet autoantibody-positive donors is tightly correlated with the presence of enteroviral capsid protein-1 (VP1), thus showing the existence of an antiviral machinery actively contributing to the islet inflammatory response during viral infections in T1D (13). Of note, antiviral signaling molecules initiating downstream inflammatory pathway activation have been previously detected in human pancreatic islets and linked to the pathogenesis of fulminant T1D (14). Indeed, the activation of antiviral signaling mechanisms is initiated by specific intracellular sensors of viral nucleic acids and/or components. Among these sensors, melanoma differentiation-associated gene 5 (MDA5) is the most important. MDA5, which belongs to the retinoic acid-inducible gene 1 (RIG-I)-like receptor (RLR) family and is encoded by the IFIH1 gene, is a dsRNA-binding protein that preferentially recognizes viral intermediates of long dsRNAs *via* interaction with its helicase domain (15). As such, MDA5 is required for intracellular immunity against several classes of viruses, including the Picornaviruses family and, consequently, the Enteroviruses genus. Once activated, MDA5 leads to the downstream recruitment of mitochondrial antiviral signaling protein (MAVS) and the subsequent triggering of a signaling cascade culminating in the activation of nuclear factor‐kappa B (NFκB), interferon‐regulating factor IRF3, and IRF7 (15). Notably, MDA5 knockdown in β-cells decreased dsRNA-induced cytokine and chemokine expression, thus limiting the inflammatory response (16).

The importance of MDA5 function in T1D pathogenesis is also highlighted by the existence of several SNPs conferring increased risk or protection from T1D (17–19). Polymorphisms conferring increased risk of T1D (e.g., A946T, TT risk genotype) have been shown to induce a weaker interferon-mediated inflammatory response in human pancreatic islets infected with Coxsackieviruses, confirming the role of MDA5 in antiviral inflammatory response and the effects of these polymorphisms on its function (20). As such, a reduced function of MDA5 may lead to a defective viral clearance and long-lasting virus persistence, thus causing a mild inflammation and potential activation of autoimmunity in genetically susceptible individuals.

Although previous studies started to investigate the role of MDA5 in β-cells (20, 21) and of its activation upon viral infection or following inflammatory stresses, both in rodents and in humans (14, 20–23), a full characterization of its expression and distribution in human pancreatic tissues both in T1D and in nonpathological conditions has not been analyzed yet in detail.

Against this background, we presently analyzed MDA5 expression distribution using the multiplex immunofluorescence technique in human pancreatic tissues obtained from nondiabetic (autoantibody-negative as well as autoantibody-positive) and T1D donors from three different organ donor biorepositories (EUnPOD, nPOD, and DiViD study), uncovering qualitative and quantitative differences in the pattern of MDA5 expression occurring in T1D vs nondiabetic pancreas.

## 2 Materials and Methods

### 2.1 Donors

Formalin-fixed and paraffin embedded (FFPE) human pancreatic sections were obtained from n=10 nondiabetic autoantibody-negative, n=2 autoantibody-positive, n=10 recent-onset, and n=2 long-standing T1D donors, belonging to three different cohorts: EUnPOD, nPOD, and DiViD. Demographical/clinical characteristics of donors are reported in **Table 1**.

**Table 1:** Main demographical/clinical characteristics of donors included in the study.

Donor Type	Case ID	Cohort	Autoantibodies	Age (years)	Gender	BMI (Kg/m^2^)	Diabetes Duration [years (y); weeks (w)]
ND	311017	EUnPOD	GADA neg, IA-2A neg, ZnT8A neg	54	F	21,3	/
ND	141117	EUnPOD	GADA neg, IA-2A neg, ZnT8A neg	49	M	25,8	/
ND	181117	EUnPOD	GADA neg, IA-2A neg, ZnT8A neg	39	M	23,6	/
ND	110118	EUnPOD	GADA neg, IA-2A neg, ZnT8A neg	46	F	32,5	/
ND	210518	EUnPOD	GADA neg, IA-2A neg, ZnT8A neg	44	M	24,5	/
ND	161118	EUnPOD	GADA neg, IA-2A neg, ZnT8A neg	39	F	22,3	/
ND	110119	EUnPOD	GADA neg, IA-2A neg, ZnT8A neg	22	M	27,2	/
ND	6179	nPOD	GADA neg, IA-2A neg, ZnT8A neg, mIAA neg	22	F	n/a	/
ND	6178	nPOD	GADA neg, IA-2A neg, ZnT8A neg, mIAA neg	25	F	n/a	/
ND	6098	nPOD	GADA neg, IA-2A neg, ZnT8A neg, mIAA neg	18	M	n/a	/
Aab+	6027	nPOD	GADA neg, IA-2A neg, ZnT8A pos, mIAA neg	19	M	n/a	/
Aab+	6167	nPOD	GADA neg, IA-2A pos, ZnT8A pos, mIAA neg	37	M	n/a	/
T1D-RO	Case 1	DiViD	IA pos, GADA pos, IA-2A pos, ZnT8A pos	25	F	21	4w
T1D-RO	Case 2	DiViD	IA pos, GADA neg, IA-2A pos, ZnT8A pos	24	M	20,9	3w
T1D-RO	Case 3	DiViD	IA pos, GADA neg, IA-2A pos, ZnT8A pos	34	F	23,7	9w
T1D-RO	Case 4	DiViD	IA pos, GADA pos, IA-2A neg, ZnT8A pos	31	M	25,6	5w
T1D-RO	Case 5	DiViD	IA pos, GADA pos, IA-2A neg, ZnT8A pos	24	F	28,6	5w
T1D-RO	Case 6	DiViD	IA pos, GADA neg, IA-2A neg, ZnT8A neg	35	M	26,7	5w
T1D-RO	6113	nPOD	GADA neg, IA-2A neg, ZnT8A neg, mIAA pos	13	F	n/a	1y
T1D-RO	6087	nPOD	GADA neg, IA-2A neg, ZnT8A pos, mIAA pos	18	M	n/a	4y
T1D-LS	060217	EUnPOD	GADA pos, IA-2A neg, ZnT8A neg	39	F	24,5	21y
T1D-LS	120718	EUnPOD	GADA neg, IA-2A neg, ZnT8A neg	81	F	31,2	45y

The following characteristics for each donor are reported: donor type (ND, nondiabetic; Aab+, autoantibody-positive; T1D-RO, type 1 diabetic recent-onset; T1D-LS, type 1 diabetic long-standing); case ID and cohort group (EUnPOD, nPOD, and DiViD); islet autoantibody-positivity; age (years); gender (F/M); body mass index; diabetes duration (years or weeks from diagnosis). n/a, not available.

#### 2.1.1 EUnPOD Cohort

Whole pancreata were obtained from brain-dead multi-organ donors within the European Network for Pancreatic Organ Donors with Diabetes (EUnPOD), in the context of the INNODIA consortium (www.innodia.eu). After acquisition of informed research consent, collected pancreata were processed using standardized procedures at the University of Pisa. INNODIA EUnPOD multiorgan donors’ pancreata were obtained with the approval of the local Ethics Committee at the University of Pisa. In this study, we analyzed pancreatic sections from 7 nondiabetic autoantibody-negative (ND) and 2 long-standing (LS) T1D donors.

#### 2.1.2 nPOD Cohort

The whole pancreas was obtained from brain-dead multi-organ donors within the Network for Pancreatic Organ Donors with Diabetes (nPOD), founded with the support of the Juvenile Diabetes Research Foundation (JDRF, www.jdrf.npod.org). After the acquisition of informed research consent, the pancreas collected was processed using standardized procedures at the University of Florida. In the present study, we used FFPE pancreatic sections from 3 nondiabetic autoantibody-negative (ND), 2 nondiabetic autoantibody positive (Aab^+^), and 2 T1D donors.

#### 2.1.3 DiViD Cohort

Following the acquisition of appropriate consents, n=6 recent-onset (< 9 weeks from diagnosis) T1D patients underwent pancreatic biopsy by adopting laparoscopic pancreatic tail resection in the context of the Diabetes Virus Detection (DiViD) study. The pancreatic tissue was processed for multiple purposes including FFPE tissue blocks. Collection of the pancreatic tissue in the DiViD study was approved by the Norwegian Governments Regional Ethics Committee. Written informed consent was obtained from all individuals with type 1 diabetes after they had received oral and written information from the diabetologist and the surgeon separately.

### 2.2 Immunofluorescence Staining

FFPE sections were analyzed using multiplexed (triple or quadruple) immunofluorescence staining followed by multicolor confocal imaging analysis and/or whole-slide scanning analysis in order to evaluate the expression and localization of melanoma differentiation-associated gene 5 (MDA5).

After deparaffinization and rehydration through a gradient ethanol series and finally distilled H_2_O, sections were incubated with Tris-buffered saline (TBS) supplemented with 5% donkey serum (for MDA5 staining) and TBS supplemented with 5% goat serum (for insulin, glucagon, somatostatin, and chromogranin-A staining) to reduce nonspecific reactions. Antigen retrieval was performed with 10 mM Tris/1 mM EDTA/0.05% Tween 20, pH 9 in boiling water for 20 min. Subsequently, sections were incubated with polyclonal goat anti-human MDA5 (dilution 1:100; ab4544, Abcam) overnight at 4°C, polyclonal guinea pig anti-insulin (undiluted, IR002, Dako) for 20 min at room temperature, monoclonal mouse anti-glucagon (dilution 1:300, MAB1249, clone #181402, R&D Systems) or monoclonal rabbit anti-glucagon (dilution 1:100, A0565, Dako) for 1 h at room temperature, monoclonal rat anti-human somatostatin (dilution 1:100; MAB2358, R&D), and monoclonal rabbit anti-chromogranin (dilution 1:400, ab15160, Abcam) incubated overnight at 4°C, as primary antibodies. After incubation with primary antibodies, sections were incubated with secondary antibodies, reacting with goat, mouse, guinea pig, rabbit, and rat [all IgG (H+L) Alexa Fluor conjugated, dilution 1:500; Molecular Probe] for 1 h or goat anti-rabbit IgG (H+L) Brilliant Violet (dilution 1:50, Jackson ImmunoResearch) for 2 h ( all at room temperature). DNA was counterstained with DAPI. Sections were finally mounted with a DAKO Fluorescence Mounting Medium (S3023 Dako). Reagent details are reported in **Supplementary Table 1**.

### 2.3 Image Acquisition and Analysis

Images were acquired using a Leica TCS SP5 confocal laser scanning microscope system (Leica Microsystems, Wetzlar, Germany) and a NanoZoomer S60 Digital slide scanner (Hamamatsu Photonics K.K., Hamamatsu City, Japan). Images were analyzed using Leica Application Advance Fluorescence (LasAF) and with the NDP view2 plus software. In particular, the percentage of colocalization rate of MDA5-insulin and MDA5-glucagon for each pancreatic islet was quantified determining the region of interest (ROI), drawn to calculate the “colocalization rate” (which indicates the extent of colocalization in percentage) as the ratio between the colocalization area and the image foreground (in which the colocalization area represents the ratio of the area of colocalizing fluorescence signals, and the image foreground represents the image area with fluorescence signal, calculated from the difference between the area of ROI and the area of the image background).

### 2.4 Statistical Analysis

Statistical analyses were performed using Graph Pad Prism 8 (GraphPad software, San Diego). Results were expressed as median with interquartile range. Differences between groups were assessed by Mann–Whitney U test or Kruskal–Wallis multiple comparison test (for nonparametric data). We considered statistically significant a p-value less than 0.05 (p <0.05).

## 3 Results

### 3.1 Immunofluorescence Analysis of Pancreatic MDA5 Expression in Nondiabetic Donors Reveals a Preferential Localization in α-Cells

Firstly, we sought to determine the expression and distribution of MDA5 in adult nondiabetic human pancreas using multiplex immunofluorescence. To this aim, we analyzed FFPE pancreatic tissue sections from n=10 nondiabetic/autoantibody-negative adult donors (age: 35.8 ± 12.9 years; gender: 5 females, 5 males) belonging to EUnPOD and nPOD networks. In nondiabetic human pancreas, MDA5 was exclusively expressed in pancreatic islet endocrine cells, as shown by a near perfect colocalization between chromogranin-A and MDA5 (**Supplementary Figure 1A, panels A**–**L**). Rare, scattered MDA5-positive, chromogranin A-positive cells were also identified interspersed within the exocrine pancreas (**Supplementary Figure 1A, panels I**–**L, yellow arrow**). In such circumstances, no MDA5-positive, chromogranin A-negative scattered cells were identified.

**Figure 1:**
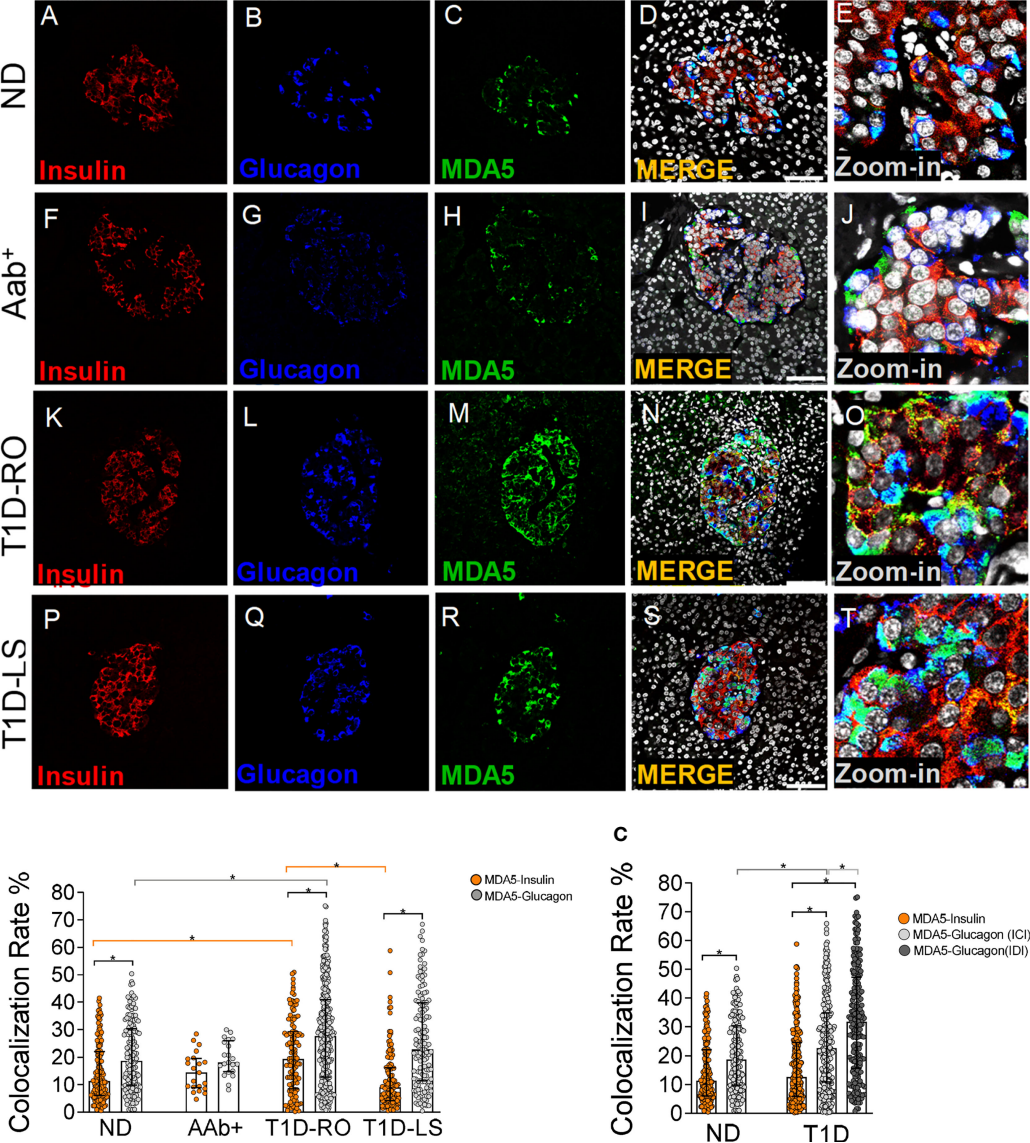
Triple immunofluorescence analysis of insulin, glucagon, and MDA5 in human pancreata of nondiabetic, Aab+, and T1D multiorgan donors. **(A)** Representative images showing fluorescence confocal microscopy imaging analysis of FFPE pancreatic tissue sections derived from nondiabetic donors (panels A–E) (n=10) (ND), autoantibody-positive donors (panels F–J) (n=2: 6027, 6167) (Aab+), type 1 diabetic recent-onset donors (panels K–O) (T1D-RO) (n=8: DiViD cases -1, -2, -3, -4, -5, -6, nPOD 6113, 6087), and type 1 diabetic long-standing donors (panels P–T) (T1D-LS) (n=2: EUnPOD 060217, 120718), stained for insulin (red, panels A, F, K, P), glucagon (blue, panels B, G, L, Q), and MDA5 (green, panels C, H, M, R). Channel merge shows the colocalization of insulin and MDA5 in yellow/orange and of glucagon and MDA5 in turquoise color. A zoom-in inset reports details of pancreatic islets of channel merge images. Scale bar in panels D, I, N, S is 75 μm. **(B)** Bar plot graphs showing the colocalization rate analysis of MDA5-insulin (orange), MDA5-glucagon (light gray) in nondiabetic controls (ND) (n=10), autoantibody-positive (Aab+) (n=2), T1D-RO (n=8), and T1D-LS (n=2). Each dot represents an individual pancreatic islet reported as a colocalization rate value between MDA5-glucagon and MDA5-insulin. Median with interquartile range values are reported as a histogram plot. Statistics performed using ANOVA followed by Kruskal–Wallis multiple comparison test (*p<0.05). **(C)** Bar plot graph showing the colocalization rate analysis of MDA5-insulin (orange), MDA5-glucagon in insulin-containing islets (ICIs) (light gray), MDA5-glucagon in insulin-deficient islets (IDIs) (dark gray) in nondiabetic controls (ND) (n=10) and in T1D donors (n=10, RO+LS). Each dot represents an individual pancreatic islet reported as a colocalization rate value of MDA5-glucagon or MDA5-insulin. Median with interquartile range values are reported. Statistics performed using ANOVA followed by Kruskal–Wallis multiple comparison test (*p<0.05).

In order to establish the endocrine cell types expressing MDA5 in nondiabetic human pancreas, we performed a triple immunofluorescence analysis aimed at detecting α- and β-cells alongside with MDA5. In human pancreatic islets, MDA5 was expressed both in α- and β-cells (**Figure 1A**, panels A–D and **Supplementary Figure 1B**). Outside pancreatic islets, most of the scattered MDA5-positive cells were also glucagon-positive and rarely showed insulin positivity. Moreover, no MDA5-positive, insulin- and glucagon-negative cells were identified in islets or interspersed in the exocrine tissue, thus confirming that in nondiabetic human pancreas, MDA5 was expressed in endocrine cells, exclusively in α- or in β-cells. Remarkably, the imaging colocalization analysis performed on a total of 147 pancreatic islets from 10 nondiabetic, autoantibody-negative adult donors showed a significantly higher colocalization rate of MDA5-glucagon in comparison to MDA5-insulin (20.5 ± 12.5% vs 14.4 ± 10.6%, p<0.01) (**Figure 1A**, panels A–D, **Figures 1B, C**).

These results indicate that (*i*) in nondiabetic human pancreas, MDA5 was expressed in endocrine cells; (*ii*) in pancreatic islets, a higher proportion of α-cells were positive for MDA5 in comparison to β-cells; and (*iii*) in such context, only a small fraction (14–20%) of β- and α-cells were positive for MDA5.

### 3.2 MDA5 Expression Is Increased in Pancreatic Islets of T1D Donors

The expression and distribution of MDA5 was also analyzed in pancreata obtained from autoantibody-positive (Aab^+^) and from T1D donors with different disease duration, namely recent-onset (RO) (3 weeks to 4 years) and long-standing (LS) (>20 years) donors. Overall, MDA5 expression pattern was analyzed in n=2 Aab^+^, n=8 T1D-RO (6 from DiViD study and 2 from nPOD cohort), and n=2 T1D-LS donors (from the EUnPOD cohort) (**Table 1**).

In line to what previously observed in nondiabetic donor pancreata, MDA5 was expressed in α- and β-cells both in Aab^+^ and T1D donors (**Figure 1A**). Such data were confirmed using a 3D *z*-scan analysis using fluorescence confocal microscopy (**Supplementary Figure 2A**), revealing the actual colocalization of MDA5 with insulin or glucagon signals, as shown by imaging analysis (**Supplementary Figure 2B**). Quadruple immunofluorescence analysis of chromogranin-A, insulin, glucagon, and MDA5 as well as quadruple immunofluorescence analysis of somatostatin, insulin, glucagon, and MDA5 in the pancreatic tissue of T1D donors (**Supplementary Figures 3A, B**) further confirmed that MDA5-positive cells were endocrine but not somatostatin-positive, thus exclusively α- or β-cells.

The colocalization rate analysis in pancreatic islets revealed a preferential MDA5 expression in α- vs β-cells also in the T1D context (27.8 ± 17.5% vs 16.3 ± 13.0%, n=246 ICI and n=224 IDI, p<0.0001), independently from disease duration (T1D RO: 28.5 ± 17% vs 20.6 ± 13.5%; T1D-LS: 26.1 ± 17.1% vs 11.9 ± 10.8%). Such pattern occurred in Aab^+^ donors as well.

Importantly, the pancreatic islets of T1D-RO donors showed a higher colocalization rate of MDA5-glucagon as well as MDA5-insulin in comparison to nondiabetic controls (**Figure 1B**) (28.5 ± 17% vs 20.5 ± 12.5% and 20.6 ± 13.5%; vs 14.4 ± 10.6%, respectively; p< 0.05). Such an increase was more pronounced in the β-cells of T1D-RO with respect to those of T1D-LS donors who showed a similar MDA5-insulin colocalization rate with respect to nondiabetic controls (**Figure 1B**).

As expected, a case-by-case analysis of MDA5-glucagon and MDA5-insulin colocalization rate values highlighted the elevated heterogeneity among islets and among different donors (**Supplementary Figure 4**), even though the increased proportion of α- and β-cells positive for MDA5 in T1D cases vs nondiabetic controls was consistent. Of note, DiViD case-3 showed the highest MDA5-insulin colocalization rate among DiViD cases (**Supplementary Figure 4**).

Interestingly, an additional analysis of MDA5-glucagon and MDA5-insulin colocalization rate in T1D-RO and T1D-LS performed taking into consideration ICIs and IDIs separately revealed that in IDIs, the colocalization rate of MDA5-glucagon was higher with respect to ICIs (**Figure 1C**).

### 3.3 Identification of MDA5-Positive/Hormone-Negative Islet-Like Structures in Pancreas of Recent-Onset T1D Donors

**Figure 2:**
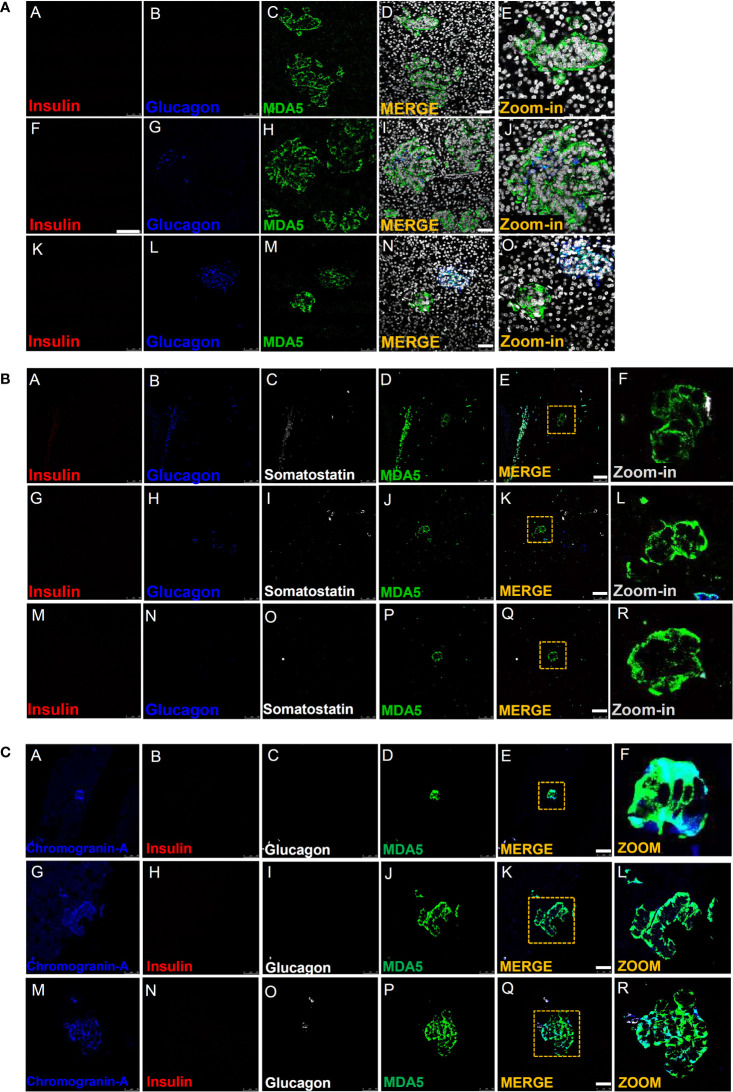
Immunofluorescence detection of MDA5-positive/hormone-negative islet-like structures in the pancreas of T1D donors. **(A)** Representative images reporting triple immunofluorescence analysis of insulin (red, panels A, F, K), glucagon (blue, panels B, G, L), MDA5 (green, panels C, H, M), DAPI (white) in T1D-RO donors (nPOD 6113: panels A–E; nPOD 6087: panels F–J; DiViD case 3: panels K–O). Zoom-in insets (panels E, J, O) report details of channel merge images. Scale bar in panels D, I, N is 50 µm. **(B)** Representative images of quadruple immunofluorescence analysis of insulin (red, panels A, G, M), glucagon (blue, panels B, H, N), somatostatin (white, panels C, I, O), and MDA5 (green, panels D, J, P) in T1D-RO donors. Zoom-in insets (F, L, R) report details of channel merge images. Scale bar in panels E, K, Q is 50 µm. **(C)** Representative images of quadruple immunofluorescence analysis of chromogranin-A (blue, panels A, G, M), insulin (red, panels B, H, N), glucagon (white, panels C, I, O), and MDA5 (green, panels D, J, P) in T1D-RO donors. Zoom-in insets (F, L, R) report details of channel merge images. Scale bar in panels E, K, Q = 50 µm.

In all T1D-RO donors [DiViD (n=6) and nPOD (n=2)], we observed several cells showing MDA5-positive signal, though negative for both insulin and glucagon; on the contrary, in nondiabetic control donor pancreas, we did not observe MDA5-positive, insulin- and glucagon-negative cells, neither within the islet parenchyma nor scattered in the exocrine (**Figure 2A**).

Interestingly, such cells were histologically organized in structures reminiscent of the islet architecture (**Figure 2A**, panel E), whose diameter and cell numbers varied consistently (**Figure 2A**, panels I, D, N). In some we observed several glucagon-positive cells, surrounded by a consistent number of MDA5-positive, insulin- and glucagon-negative cells (**Figure 2A**, panels L, J), potentially suggesting an islet lineage.

A *z*-scan confocal imaging analysis further confirmed that such structures were MDA5-positive but insulin- and glucagon-negative, also upon 3D scanning imaging and a deconvolution projection analysis (**Supplementary Figures 5A, B**).

Strikingly, MDA5-positive cells belonging to such structures were somatostatin-negative (**Figure 2B**, panels A–R) but clearly positive for chromogranin A, thus definitely suggesting their endocrine origin (**Figure 2C**, panels A–R). In addition, in such structures, the MDA5 fluorescence signal was visibly higher than what was previously observed for α- or β-cells, thus suggesting an elevated expression of MDA5 in these cells.

Finally, a detailed observation of pancreatic tissue sections stained for insulin, glucagon, and MDA5 revealed that such structures were present exclusively in T1D-RO donors (both DiViD and nPOD) but absent in T1D-LS (EUnPOD) or in nondiabetic control donors (both EUnPOD and nPOD).

### 3.4 Distribution Analysis of MDA5-Positive Islets in Pancreas of T1D DiViD Donors

**Figure 3:**
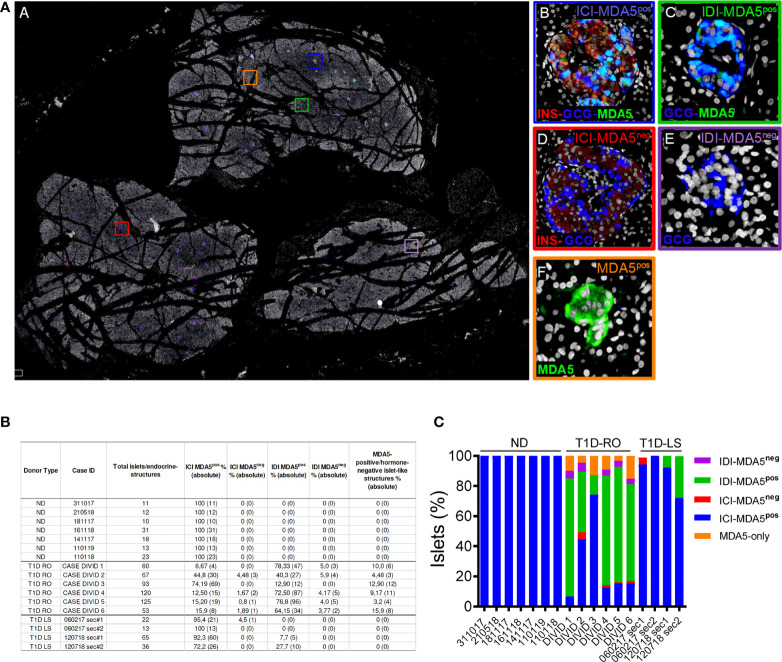
Whole-slide imaging islet distribution analysis based on MDA5 positivity in ND and T1D donors. **(A)** Representative whole-slide image of T1D case-3 (DiViD) of triple immunofluorescence staining for insulin (INS, red), glucagon (GCG, blue), MDA5 (green), and DAPI (white) analyzed using a Nanozoomer S60 slide scanner. The image (panel A) reports the localization of 5 different histological structures identified in T1D pancreatic sections. Zoom-in insets for each type of islet or islet-like structure are reported on the left. Blue square (panel B): insulin-containing islet (ICI)-MDA5 positive; red square (panel D): insulin-containing islet-MDA5 negative; green square (panel C): insulin-deficient islet (IDI)-MDA5 positive; magenta square (panel E): insulin-deficient islet-MDA5 negative; orange square: MDA5-only positive islet-like structures. **(B)** Table showing islet distribution based on MDA5 positivity in pancreatic tissue sections of ND, T1D-Recent Onset (RO), and T1D-Long Standing (LS). Results are reported as percentage values (alongside with absolute values) over the total number of islets detected. Donor types as well as case IDs are reported. **(C)** Graphical representation of the distribution of pancreatic islets based on MDA5 positivity in pancreatic tissue sections of ND, T1D-RO (DiViD cases), and T1D-LS.

In order to evaluate the distribution and eventual heterogeneity of pancreatic islets in terms of MDA5 expression, we performed a whole slide scanning imaging analysis of pancreatic tissue sections derived from EUnPOD nondiabetic donors and from T1D DiViD cases, stained for insulin, glucagon, and MDA5 (**Figure 3A**, panel A). Whole slide fluorescence scanning imaging analysis has the advantage to allow the exploration of the entire pancreatic tissue section.

In nondiabetic donors, ICIs were all positive for MDA5. No MDA5-positive/hormone-negative islet-like structures were identified in the nondiabetic context, confirming what was previously observed by confocal microscopy.

In T1D DiViD donors, we found a high heterogeneity among pancreatic islets, including the presence of ICIs or IDIs-MDA5 negative (**Figure 3A**, panels C, D). Overall, in T1D DiViD cases, a large fraction of pancreatic islets were positive for MDA5, and only a minor proportion of them showed very low or null expression of MDA5 (**Figures 3B, C**). In DiViD cases, a variable percentage of MDA5-chromogranin A-positive/hormone-negative islet-like structures (over the total number of islets detected) were identified, ranging from 3.2% (DiViD case-5) to 15.9% (DiViD case-6) (**Figure 3B**).

Collectively, these results suggest that (*i*) in T1D donors, human pancreatic islets show a higher proportion of α- and β-cells expressing MDA5 with respect to nondiabetic controls or T1D-LS donors; (*ii*) in T1D, both α- and β-cells drive the increase in MDA5-positive cells in human pancreatic islets; (*iii*) such an increase is more pronounced in T1D-RO with respect to T1D-LS; and (iv) in T1D-RO donors, we uncovered the existence of MDA5-expressing islet-like structures, which were positive for chromogranin A but negative for islet main hormones (insulin, glucagon, and somatostatin).

## 4 Discussion

The hypothesis that viruses could play a key role in the pathogenesis of T1D has a long history. Enteroviruses (EVs), especially the Coxsackievirus B-group (CVB), have been associated to the initiation and/or acceleration of pancreatic islet autoimmunity (24). As a matter of fact, many evidence have shown the ability of EVs to infect human pancreatic β-cells and to impair their function and/or survival (4–8). Pancreatic immunohistochemical data showed the selective positivity of viral capsid protein-1 (VP1) in β-cells. Such selectivity is corroborated by additional evidence showing (*i*) a strong permissiveness of β-cells to EVs when infected *in vitro* (25, 26) and (*ii*) the specific expression of Coxsackie and adenovirus receptor (CAR) in β-cells (12). Of note, although a strong causal association between EV infections and T1D is still debated, several mechanisms have been proposed, including (i) direct destruction of β-cells, (ii) by-stander activation of autoreactive T cells, and (iii) molecular mimicry and viral persistence causing β-cell dysfunction.

A crucial piece of the puzzle is represented by intracellular antiviral molecules involved in innate immune response, which characterize the first line of defense by coordinating signaling pathway activation leading to inflammatory responses. Among others, MDA5 has been identified as an important component of the intracellular innate immune response to EV intermediate dsRNAs, which is necessary to prevent early replication of the virus and to limit tissue damage through a rapid and efficient pathogen clearance (21). However, a massive and excessive inflammatory response triggered by MDA5 as a result of a severe enteroviral infection can also cause significant tissue damage and further activate other inflammatory signaling pathways, as described in fulminant diabetes (14, 27, 28).

Although MDA5 expression and distribution have been evaluated in murine pancreas and in pancreatic samples of multiorgan donors affected by fulminant diabetes, a detailed characterization of MDA5 expression in human T1D and nondiabetic pancreas is still missing. We took advantage from several organ biorepository networks in order to analyze the MDA5 expression pattern in pancreatic tissues from T1D donors with different disease duration, as well as from nondiabetic/autoantibody-positive and autoantibody-negative donors. This has been accomplished by accessing three different biorepositories: EUnPOD, nPOD, and DiViD study biobank.

Firstly, we analyzed the expression pattern of MDA5 in the pancreas of control donors. In nondiabetic pancreas, we showed that MDA5 was specifically expressed in endocrine cells, particularly in α- and in β-cells, thus confirming that islet cells are equipped to efficiently respond to viral RNA intermediates. Of interest, in a nondiabetic context, a higher proportion of α-cells expressed MDA5 with respect to β-cells. Such results are concordant with different bulk RNA transcriptomics datasets of sorted human α- and β-cells (22, 29, 30) showing a preferential expression of MDA5 mRNA in α-cells with respect to β-cells; additionally, these data validate the results obtained through the MDA5 antibody used in the present study.

The colocalization rate of MDA5-glucagon and MDA5-insulin in nondiabetic controls was highly heterogenous both among islets of the same donor and among different donors; nevertheless, the overall result showed a significant preferential localization in α-cells. Such heterogeneity was expected based on previous findings on other molecules (31, 32) and on other reports (33, 34).

The preferential localization of MDA5 in α-cells suggests that this cell type is better equipped than β-cells to efficiently respond to EV entry by promptly activating pro-inflammatory signaling pathways, which contribute to virus clearance in the early stages of infection. Such observation is substantiated by *in vitro* studies showing a higher capacity of α-cells vs β-cells to increase the expression of MDA5 and of other key antiviral proteins (e.g., STAT1 and MX1) in response to CVB infection (21). As a matter of fact, MDA5 has been found to play a key role in IFNs-I and IFNs-III induction and in inflammatory cytokines production following virus infection; consequently, synthesis of pro-inflammatory mediators may lead to the expression of ISGs, whose products direct antiviral and immunoregulatory effects. On the contrary, lack of a fully functional antiviral signaling machinery characterized by MDA5 loss of function, absence, or reduced expression could be linked to the establishment of a subtle chronic inflammatory response and to a long-lasting noncytolytic infection, which may activate autoimmunity.

Our results also showed a higher proportion of α- and β-cells expressing MDA5 in recent-onset T1D cases with respect to nondiabetic controls. The increased colocalization rate between MDA5-glucagon and MDA5-insulin in T1D cases possibly reflects ongoing inflammation of pancreatic islets, particularly in recent-onset cases. Of note, the strongest MDA5 increase was observed in the β-cells of T1D DiViD case-3, which showed a high level of insulitis alongside with a strong expression of HLA class-I molecules and several specific cytokines and chemokines (31, 35, 36). These results are in line with *in vitro* studies showing that increased expression of MDA5 is also driven by pro-inflammatory molecules. Of note, it has been demonstrated that exposure of pancreatic islet cells to viruses or to viral intermediates (dsRNAs) leads to MDA5 increased expression (16, 20); remarkably, evidence of EV infection has been detected in the pancreatic islets of all DiViD cases across different laboratories employing multiple approaches (6, 10).

An additional result worth mentioning is the higher colocalization rate of MDA5-glucagon in IDIs vs ICIs. Based on the fact that IDIs are deprived of β-cells, thus less inflamed than ICIs, we can hypothesize that the increase in MDA5 in IDIs could be due to other factors than pro-inflammatory molecules activity. It has been reported that in specific conditions, MDA5 could also sense endogenous dsRNAs generated by epigenetic activation of repeated genomic DNA sequences. Indeed, increased MDA5 expression and activation has been reported in cancers treated with demethylating agents, leading to the accumulation of endogenous RNAs (37, 38). Physiologically, this phenomenon could be due to phenotypic changes requiring the activation of epigenetic mechanisms and RNA editing modifications. As such, we can hypothesize that residual α-cells within IDIs are subjected to strong epigenetic pressure with the aim of modifying their phenotype, thus leading to the transcription of dsRNAs or to the modifications of RNA editing mechanisms, which finally increase MDA5 expression and activity. Importantly, it has been recently shown that rodent pancreatic α-cells undergo massive transcriptional changes upon total β-cell ablation, including the upregulation of genes involved in interferon signaling and proliferation (39); of note, an increased expression of MDA5 as early as 5 days post-β-cell ablation has been reported as well (39) (see GEO dataset GSE155519).

Strikingly, for the first time, we also detected several islet-like cell clusters that were positive for MDA5 but negative for the main islet hormones (insulin, glucagon, and somatostatin). Such cells were of endocrine origin since they showed positivity for chromogranin-A. Although we did not test for other minor hormones (pancreatic-polypeptide, ghrelin), the elevated number of cells composing these islet-like clusters allowed us to exclude that they could be δ- or ϵ-cells. In addition, given their morphology, structure, nuclei conformation, and, more importantly, the presently shown positivity for chromogranin-A, we can exclude their immune origin.

Increased frequency of chromogranin-A-positive/hormone-negative cells has been documented in the pancreas of rodent models with diabetes, in donors with T1D and T2D (40–42), as well as in human pancreatic tissues derived from donors affected by pancreatitis (43). Here we showed that such chromogranin-A-positive/hormone-negative cell clusters were also strongly positive for MDA5. The exact origin of these islet-like clusters has yet to be elucidated. In line to what was previously suggested, we can hypothesize that these clusters could be derived from pancreatic islet cells undergoing dedifferentiation phenomena. Interestingly, it has been shown that human β-cells dedifferentiate upon virus-like infection simulated *in vitro* by PolyI:C (PIC) treatment (44); of note, upon PIC exposure, MDA5 levels are increased alongside with a reduced expression of several genes related to β-cell identity and function (44). Similarly, it has been shown that pro-inflammatory molecules may induce β-cell dysfunction and phenotype loss, alongside with MDA5 increase. Among pro-inflammatory molecules tested, IL-1β was shown to be the most potent inducer of *in vitro* dedifferentiation (45). In support of a role of inflammation in promoting islet dedifferentiation and appearance of MDA5-positive/hormone-negative endocrine cells, we observed that one of the highest frequencies of MDA5-positive/hormone-negative islet-like clusters was detected in the pancreas of T1D DiViD case-3, which also showed a high rate of inflammation, thus corroborating the current view of an inflammation-driven mechanism of dedifferentiation induction. Strikingly, MDA5-positive/hormone-negative islet-like clusters were not observed in autoantibody-positive nondiabetic donors and in long-standing T1D donors, usually characterized by lower levels of inflammation with respect to recent-onset donors (46).

Alternatively, we can speculate that MDA5-positive/hormone-negative endocrine cells may represent newly forming islets, recapitulating what was observed in human neonatal pancreas enriched in chromogranin-A-positive/hormone-negative structures.

Whatever the origin of these islet-like clusters, a function for MDA5 in putatively undifferentiated cells should be deciphered. It is possible that high MDA5 expression found in these clusters may be the consequence of inflammatory phenomena leading to the activation of specific innate signaling pathways and, in parallel, causing dedifferentiation. On the other hand, MDA5 increased expression and activity could also be due to epigenetic changes occurring during phenotypic modifications taking place throughout dedifferentiation, causing RNA accumulation and sensing by MDA5.

One advantage of our study is the analysis of pancreatic FFPE samples belonging to multiple tissue repositories (EUnPOD, DiViD, and nPOD). However, we acknowledge that the low number of nondiabetic autoantibody-positive and T1D long-standing donors may have limited the total number of islets considered. Therefore, even though our data are statistically significant, MDA5 expression distribution should be evaluated in a larger group of nondiabetic Aab+ and T1D long-standing donors.

In conclusion, we showed that MDA5 is expressed in pancreatic endocrine cells with a preferential localization in α-cells vs β-cells, remarkably suggesting that α-cells are better equipped with respect to β-cells to respond and activate viral clearance mechanisms; such difference may render β-cells more susceptible to the establishment of persistent low-grade viral infections. We also showed that MDA5 is increased in recent-onset T1D donors possibly in consequence of elevated inflammatory conditions. Of note, in these donors, we highlighted the identification of MDA5-positive/hormone-negative endocrine cell clusters, thus opening to novel roles for MDA5 in putative dedifferentiation mechanisms in T1D.

## Data Availability Statement

The original contributions presented in the study are included in the article/**Supplementary Material**. Further inquiries can be directed to the corresponding author.

## Ethics Statement

Collection of pancreatic tissue in the DiViD study was approved by the Norwegian Governments Regional Ethics Committee. Written informed consent was obtained from all individuals with type 1 diabetes after they had received oral and written information from the diabetologist and the surgeon separately. EUnPOD: the studies involving human participants were reviewed and approved by local ethics committee of the University of Pisa (Italy). Pancreata not suitable for organ transplantation were obtained with informed written consent by organ donors’ next-of-kin and processed with the approval of the local ethics committee. The patients/participants provided their written informed consent to participate in this study.

## Author Contributions

LN, NB, and GS performed the experiments, analyzed the data, and wrote the manuscript. GG, DF, and GL analyzed the data and contributed to the scientific discussion. CF contributed to the scientific discussion. LN, GS, and FD reviewed the manuscript and designed the experiments. LK and KJ provided support for the DiViD cohort and contributed to the scientific discussion. LM and PM reviewed the manuscript, provided support for EUnPOD donors, and contributed to the scientific discussion. All authors contributed to the article and approved the submitted version.

## Funding

The work is supported by the Innovative Medicines Initiative 2 (IMI2) Joint Undertaking under grant agreement No.115797-INNODIA and No.945268 INNODIA HARVEST. This joint undertaking receives support from the Union’s Horizon 2020 research and innovation program and EFPIA, JDRF, and the Leona M. and Harry B. Helmsley Charitable Trust. FD was supported by the Italian Ministry of University and Research (2017KAM2R5_003). GS was supported by the Italian Ministry of University and Research (201793XZ5A_006).

## Conflict of Interest

The authors declare that the research was conducted in the absence of any commercial or financial relationships that could be construed as a potential conflict of interest.

## Publisher’s Note

All claims expressed in this article are solely those of the authors and do not necessarily represent those of their affiliated organizations, or those of the publisher, the editors and the reviewers. Any product that may be evaluated in this article, or claim that may be made by its manufacturer, is not guaranteed or endorsed by the publisher.
